# Changes to the core and flanking sequences of G‐box elements lead to increases and decreases in gene expression in both native and synthetic soybean promoters

**DOI:** 10.1111/pbi.13010

**Published:** 2018-09-24

**Authors:** Ning Zhang, Leah K. McHale, John J. Finer

**Affiliations:** ^1^ Department of Horticulture and Crop Science The Ohio State University Wooster OH USA; ^2^ Department of Horticulture and Crop Science The Ohio State University Columbus OH USA; ^3^ Present address: Boyce Thompson Institute Cornell University Ithaca NY USA

**Keywords:** *cis*‐regulatory element, G‐box, flanking sequences, synthetic promoter, elongation factor‐1 α promoter, glycinin promoter

## Abstract

*Cis*‐regulatory elements in promoters are major determinants of binding specificity of transcription factors (TFs) for transcriptional regulation. To improve our understanding of how these short DNA sequences regulate gene expression, synthetic promoters consisting of both classical (CACGTG) and variant G‐box core sequences along with different flanking sequences derived from the promoters of three different highly expressing soybean genes, were constructed and used to regulate a *green fluorescent protein* (*gfp*) gene. Use of the classical 6‐bp G‐box provided information on the base level of GFP expression while modifications to the 2–4 flanking bases on either side of the G‐box influenced the intensity of gene expression in both transiently transformed lima bean cotyledons and stably transformed soybean hairy roots. The proximal 2‐bp sequences on either flank of the G‐box significantly affected G‐box activity, while the distal 2‐bp flanking nucleotides also influenced gene expression albeit with a decreasing effect. Manipulation of the upstream 2‐ to 4‐bp flanking sequence of a G‐box variant (GACGTG), found in the proximal region of a relatively weak soybean glycinin promoter, significantly enhanced promoter activity using both transient and stable expression assays, if the G‐box variant was first converted into a classical G‐box (CACGTG). In addition to increasing our understanding of regulatory element composition and structure, this study shows that minimal targeted changes in native promoter sequences can lead to enhanced gene expression, and suggests that genome editing of the promoter region can result in useful and predictable changes in native gene expression.

## Introduction

*Cis*‐regulatory elements in promoters, introns or enhancers provide specific binding sites for corresponding transcription factors (TFs), which regulate the transcription of gene expression (Hernandez‐Garcia and Finer, 2014). With the increasing availability of plant genomic sequences (Brenchley *et al*., 2012; Schmutz *et al*., 2010; Schnable *et al*., 2009) and the development of bioinformatics tools (Higo *et al*., 1999; Lescot *et al*., 2002; Lichtenberg *et al*., 2010; Timothy *et al*., 2009), putative regulatory elements can be identified within targeted DNA regulatory sequences. However, the use of current online element databases (Higo *et al*., 1999; Lescot *et al*., 2002) or sequence‐driven algorithms (Lichtenberg *et al*., 2010; Timothy *et al*., 2009) frequently leads to the over‐identification of regulatory elements, most of which are not functional in vivo for transcription factor binding (Hardison and Taylor, 2012) or gene regulation. Validation of those promoters and their regulatory elements using transgene expression assays and nucleotide mutagenesis is essential to fully understand their functionality (Hernandez‐Garcia and Finer, 2014). Synthetic promoters, containing unique arrangements of regulatory elements, along with variant regulatory element sequences, have been used to study regulatory element functionality (Liu and Stewart, 2016; Mehrotra *et al*., 2017; Venter, 2007).

The G‐box element is an abundant, well‐characterized, multifunctional plant promoter regulatory element. The G‐box, first identified as ‘TCTTA*CACGTG*GCAYY’ in the promoter region of a light‐regulated gene encoding the small subunit of ribulose 1,5‐bisphosphate carboxylase/oxygenase (*RBCS*) (Giuliano *et al*., 1988), was also identified from a multitude of biotic/abiotic stress‐regulated genes including the ethylene‐inducible *PRB‐1b* gene of tobacco (Sessa *et al*., 1995), the auxin‐regulated *GmAux28* gene of soybean (Nagao *et al*., 1993), the ABA‐responsive *HVA22* gene of barley (Shen and Ho, 1995), the light‐regulated *chalcone synthase* gene of parsley (Block *et al*., 1990), the *rbcS‐1A* gene of Arabidopsis (Donald and Cashmore, 1990), the methyl jasmonate‐responsive *proteinase inhibitor II* gene of potato (Kim *et al*., 1992) and the *NtPMT1a* gene of tobacco (Xu and Timko, 2004). In most cases, G‐boxes interacted with other non‐conserved *cis*‐acting elements to co‐regulate gene expression under inducible conditions (Block *et al*., 1990; Donald and Cashmore, 1990; Faktor *et al*., 1997; Sessa *et al*., 1995; Shen and Ho, 1995; Xu and Timko, 2004). Spacing between the G‐box and the coupling element influenced the inducibility and strength of the G‐box (de Vetten and Ferl, 1994). In addition to inducible expression, G‐box elements also conferred high‐level constitutive expression (Ishige *et al*., 1999; McKendree and Ferl, 1992), or tissue‐specific gene expression (Faktor *et al*., 1997; Kobayashi *et al*., 2012).

A classical G‐box core element consists of a 6‐bp DNA sequence ‘CACGTG’ (Menkens *et al*., 1995) with variations in the first and last nucleotides, which is minimally needed to recruit G‐box‐binding factors (GBFs) (Menkens *et al*., 1995) such as basic leucine zipper (bZIP) proteins (Siberil *et al*., 2001) or basic helix‐loop‐helix (bHLH) proteins (Heim *et al*., 2003). The flanking sequences, especially the 2‐bp proximal nucleotides on both sides of the G‐box, also affected the binding specificity and affinity of GBFs (Williams *et al*., 1992). Although G‐boxes with different 2‐bp proximal flanking sequences have generated and showed different expression patterns (Ishige *et al*., 1999), modulation of gene expression by targeted modification to the flanking sequences of G‐boxes using either native or synthetic plant promoters has not been investigated.

We previously reported the isolation and characterization of twenty highly‐expressing ‘GmScream’ promoters from soybean (Zhang *et al*., 2015), from which several G‐box‐like elements were identified. In this study, we used synthetic promoters consisting of a classical 6‐bp G‐box (CACGTG) along with different flanking sequences, to evaluate changes in gene expression caused by changing the 2–4 bp flanking sequences of the G‐box. We found that a G‐box could drive very high levels of gene expression only if changes were made to its flanking nucleotides. In addition, manipulation of the 4‐bp flanking nucleotides of a naturally occurring G‐box variant in the proximal region of a relatively weak soybean glycinin promoter (GmScream3; *Glyma.10 g037100*; Zhang *et al*., 2015) led to a significant increase in gene expression, suggesting that precise changes in native promoter sequences may lead to large increases in native gene expression. These results indicate that genome editing strategies targeted to regulatory elements in promoter regions may be used to precisely modulate gene expression in a predictable manner.

## Results

### Several G‐box element fragments from GmScream promoters conferred high gene expression

**Figure 1:**
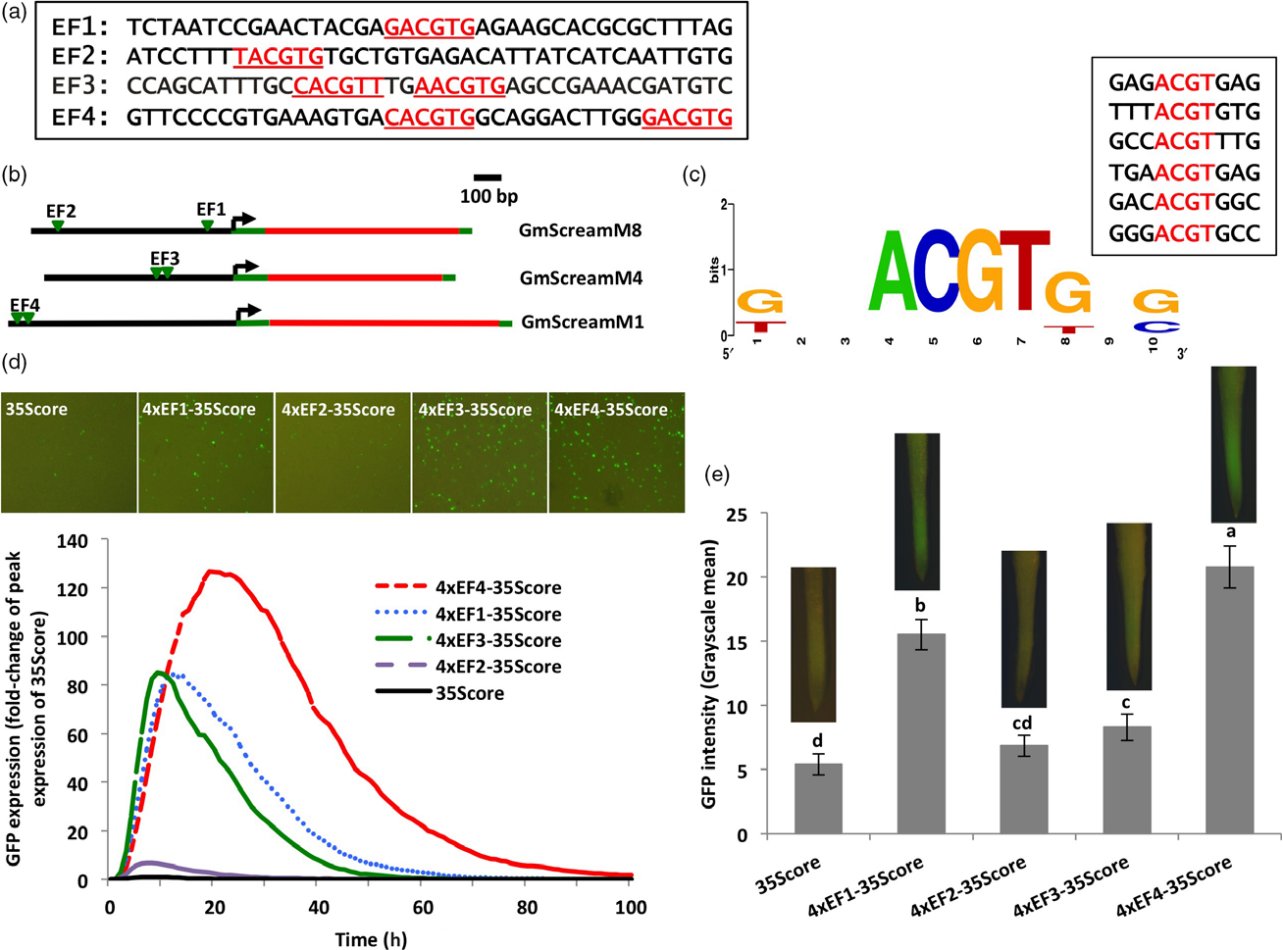
Validation of putative G‐box elements present in three strong GmScream promoters. (a) DNA fragments containing G‐box elements (Underline: G‐box elements identified using PlantCARE). (b) Position localization of G‐box elements in each GmScream promoter. (c) Sequence logo of G‐boxes generated using WebLogo (Crooks *et al*., 2004). (d) Profiles of transient GFP expression driven by fragment tetramers containing G‐box elements fused to the 35S core promoter in lima bean cotyledons over 100 h. Levels of GFP expression of each construct were reported as fold‐change of peak expression of the 35S core promoter. (e) Mean GFP expression intensity (±SE) in soybean hairy roots transformed with different synthetic element constructs. Columns followed by the same letter are not significantly different by the *t* test (LSD) at *P* < 0.05.

To further study the effect of potential *cis*‐elements on the high activity of several GmScream promoters (GmScreamM1, GmScreamM4, and GmScreamM8; Zhang *et al*., 2015), we identified G‐box‐like elements in the proximal, middle and distal regions of these promoters. The GmScreamM1, GmScreamM4 and GmScreamM8 promoters each contained two putative G‐box‐like elements. The two G‐boxes in GmScreamM1 and GmScreamM4 were close to each other while the two G‐boxes in GmScreamM8 were more widely spaced apart (Figure 1a–c). Four 40‐bp element fragments, named EF1–EF4, containing the putative G‐boxes and native flanking sequences, were selected for more detailed analysis of their contribution to gene expression. While EF1 and EF2 (from the GmScreamM8 promoter) contained only one putative G‐box, EF3 and EF4 (from the GmScreamM4 and GmScreamM1 promoters respectively) each contained two putative G‐boxes as the G‐box sequences were close to each other. Of the four G‐box‐containing fragments, EF1 and EF3 were located near (−154 and −314 respectively) the transcription start site (TSS) while EF2 and EF4 were further upstream (−727 and −869 respectively) (Figure 1b).

When tetramers of these four fragments were assembled and placed upstream of the Cauliflower Mosaic Virus 35S (35S) core promoter, and the synthetic promoter constructs were introduced into lima bean cotyledonary tissues, they all gave increased levels of transient gene expression as compared to the 46‐bp 35S core promoter (Figure 1d). Among the four G‐box‐containing constructs, the 4xEF4‐35Score drove the highest level of GFP expression, with over 120‐fold higher expression compared to the 35S core promoter (Figure 1d). Both 4xEF1‐35Score and 4xEF3‐35Score showed lower activity than the 4xEF4‐35Score, but they still drove GFP expression 80‐fold higher than the 35S core promoter (Figure 1d). The 4xEF2‐35Score showed relatively weak activity, with expression only 6.7‐fold higher than the 35S core promoter (Figure 1d). Evaluation of the four G‐box‐containing fragments in stably transformed soybean hairy roots showed results which were consistent with those obtained from transient expression analysis. The 4xEF4‐35Score construct showed the highest activity, which was significantly higher than the other element‐containing constructs (Figure 1e). The other two constructs, 4xEF1‐35Score and 4xEF3‐35Score, also drove a significantly higher GFP expression than the 35S core promoter, while 4xEF2‐35Score was not significantly different from the 35S core promoter (Figure 1e). In all, the EF4 fragment (4xEF4‐35Score) drove the highest GFP expression using both transient and stable expression analyses, and the putative G‐boxes associated with this fragment were therefore selected for further evaluation.

### The classical G‐box element in EF4 conferred high gene expression

**Figure 2:**
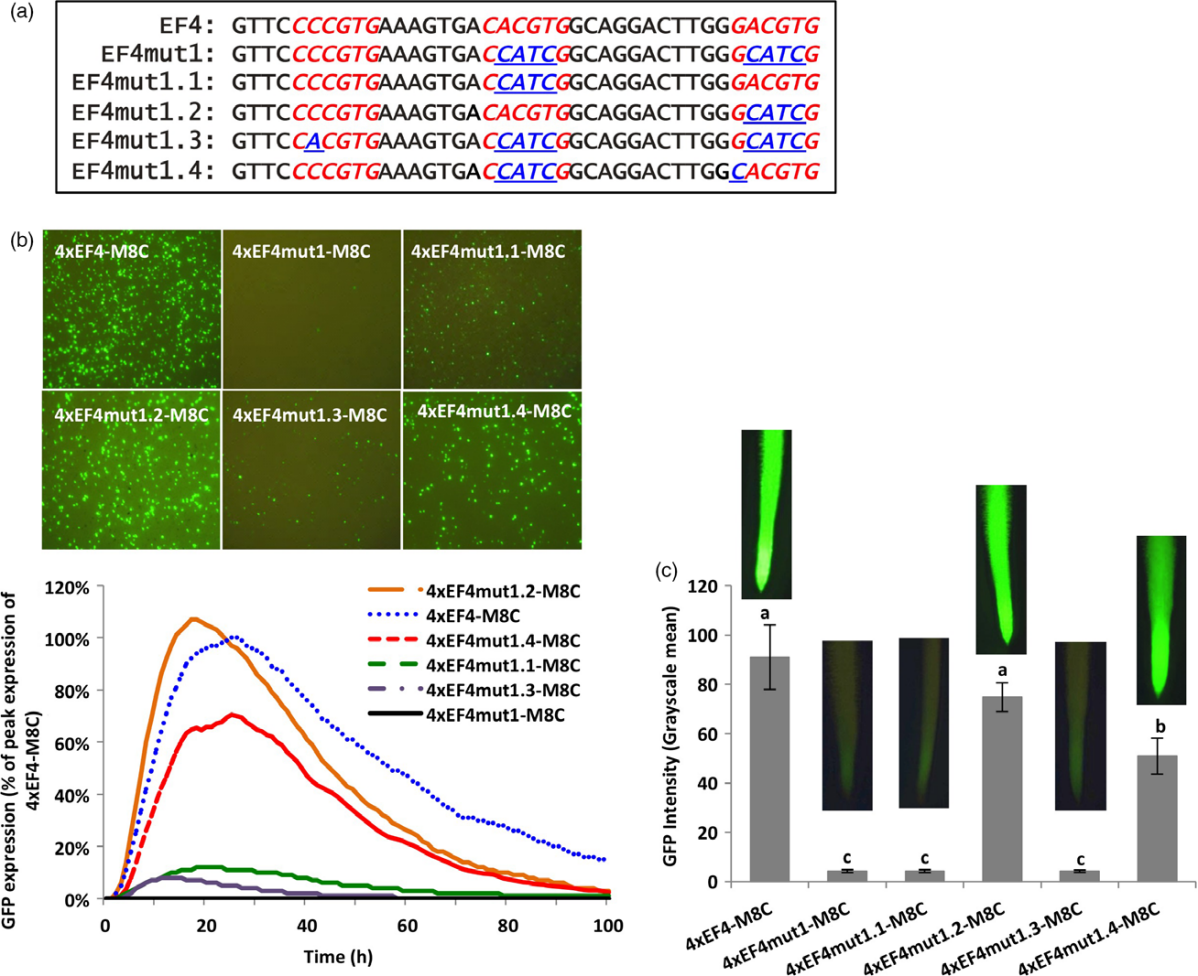
Effects of G‐box core elements on gene expression. (a) Mutagenesis of G‐box elements (Underline). (b) Profiles of transient GFP expression driven by different EF4 mutants in lima bean cotyledons over 100 h. Levels of GFP expression of each construct were reported as percentage of peak expression of the 4xEF4‐M8C. (c) Mean GFP expression intensity (±SE) in soybean hairy roots transformed with different EF4 mutants. Columns followed by the same letter are not significantly different by the *t* test (LSD) at *P* < 0.05.

The EF4 fragment contained two 6‐bp putative G‐box elements. One (CACGTG) exactly matched the classical G‐box sequence, while the other (*G*ACGTG) mismatched the classical G‐box by one nucleotide. Another 6‐bp DNA sequence (C*C*CGTG) in EF4 (Figure 2a) was not annotated as a G‐box, but also showed only one mismatch to the classical G‐box. To investigate the contribution of each putative G‐box to the high activity of the EF4 fragment, as well as explore the possibility of enhancing gene expression by changing one nucleotide to create a classical G‐box sequence, EF4 mutants containing variant G‐box forms were generated (Figure 2a). Synthetic promoters consisting of various tetrameric EF4 mutant fragments were first evaluated using transient expression in lima bean cotyledons (Figure 2b). Mutation of both ‘CACGTG’ and ‘GACGTG’ into non‐functional sequences (4xEF4mut1‐M8C) led to a dramatic decrease of GFP expression compared to the 4xEF4‐M8C control (Figure 2b). Mutation of only ‘CACGTG’ (4xEF4mut1.1‐M8C) in the EF4 fragment also significantly decreased the level of GFP expression, while mutation of only the G‐box variant ‘GACGTG’ (4xEF4mut1.2‐M8C) did not affect the intensity of GFP expression, relative to the native sequence (Figure 2b). In 4xEF4mut1.3‐M8C, where ‘CACGTG’ and ‘GACGTG’ were both mutated into non‐functional regulatory sequences, an additional modification of ‘CCCGTG’ to generate the classical G‐box ‘CACGTG’ did not give rise to high levels of GFP expression (Figure 2b). Introduction of the 4xEF4mut1.4‐M8C construct, in which the classical G‐box was made non‐functional and the ‘GACGTG’ was mutagenized into the classical G‐box ‘CACGTG’, showed significantly higher GFP expression compared to 4xEF4mut1.1‐M8C (Figure 2b). Consistent with transient expression in lima bean cotyledonary tissues, in stably transformed soybean hairy roots, 4xEF4mut1.2‐M8C showed comparable levels of GFP expression to the control 4xEF4‐M8C, while 4xEF4mut1‐M8C, 4xEF4mut1.1‐M8C and 4xEF4mut1.3‐M8C displayed significantly weaker expression (Figure 2c). The 4xEF4mut1.4‐M8C drove a significantly higher level of GFP expression compared to 4xEF4mut1.1‐M8C, but still lower expression than the control 4xEF4‐M8C (Figure 2c). In all, the native classical G‐box (CACGTG) in EF4 significantly contributed to high GFP expression, while the native G‐box variant (GACGTG) has little effect, unless it was first converted into a classical G‐box sequence. The recreated classical G‐box from the ‘CCCGTG’ sequence in EF4 did not restore high levels of GFP expression using both transient and stable expression analyses.

### Flanking sequences of the G‐box element affected gene expression

**Figure 3:**
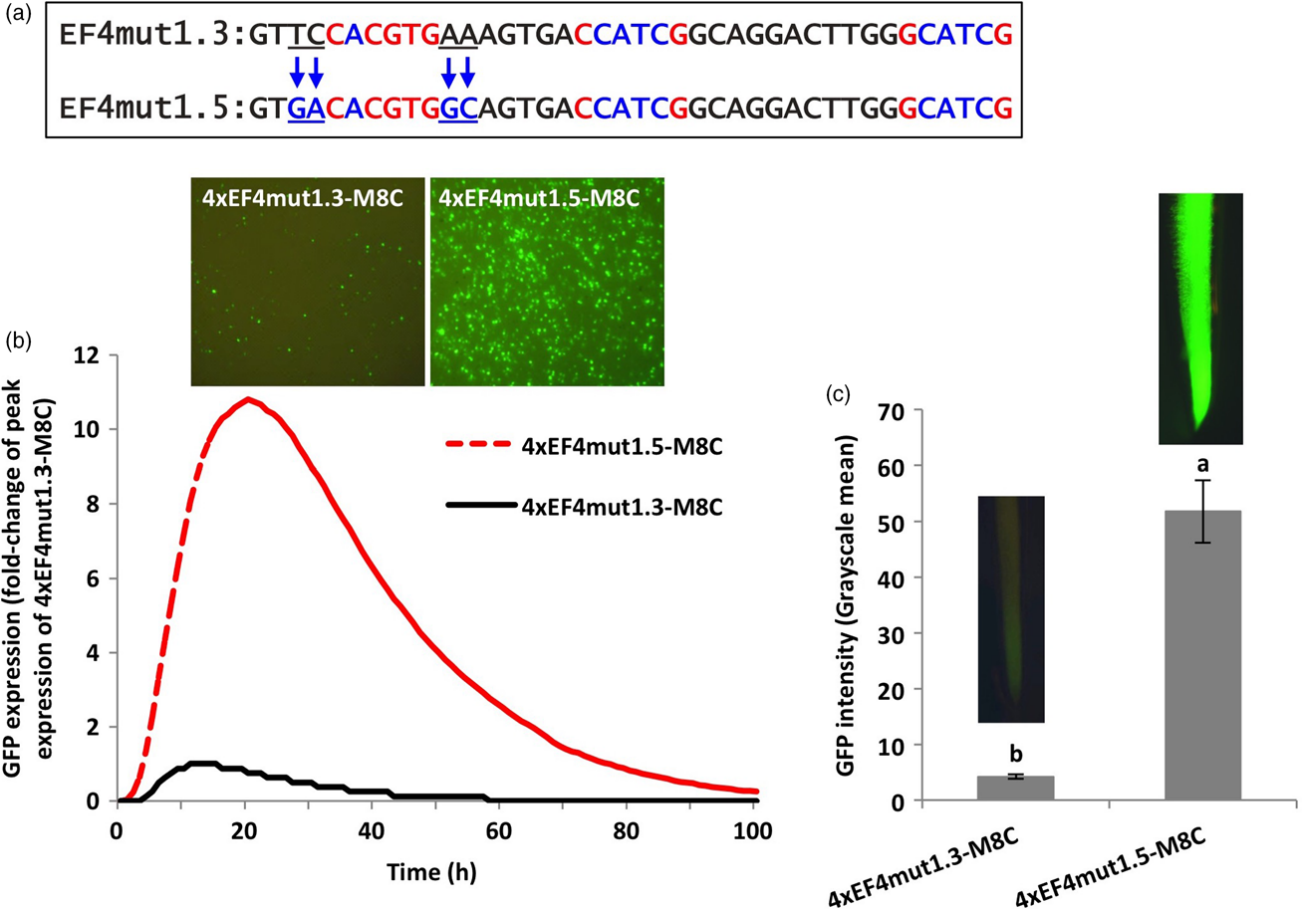
Manipulation of 2‐bp proximal flanks at both sides of a G‐box. (a) Mutagenesis of 2‐bp flanking sequences at both sides of the canonical G‐box (CACGTG). (b) Profiles of transient GFP expression in lima bean cotyledon tissues over 100 h. (c) Mean GFP expression intensity (±SE) in soybean hairy roots transformed with different constructs. Columns followed by the same letter are not significantly different by the *t* test (LSD) at *P* < 0.05.

**Figure 4:**
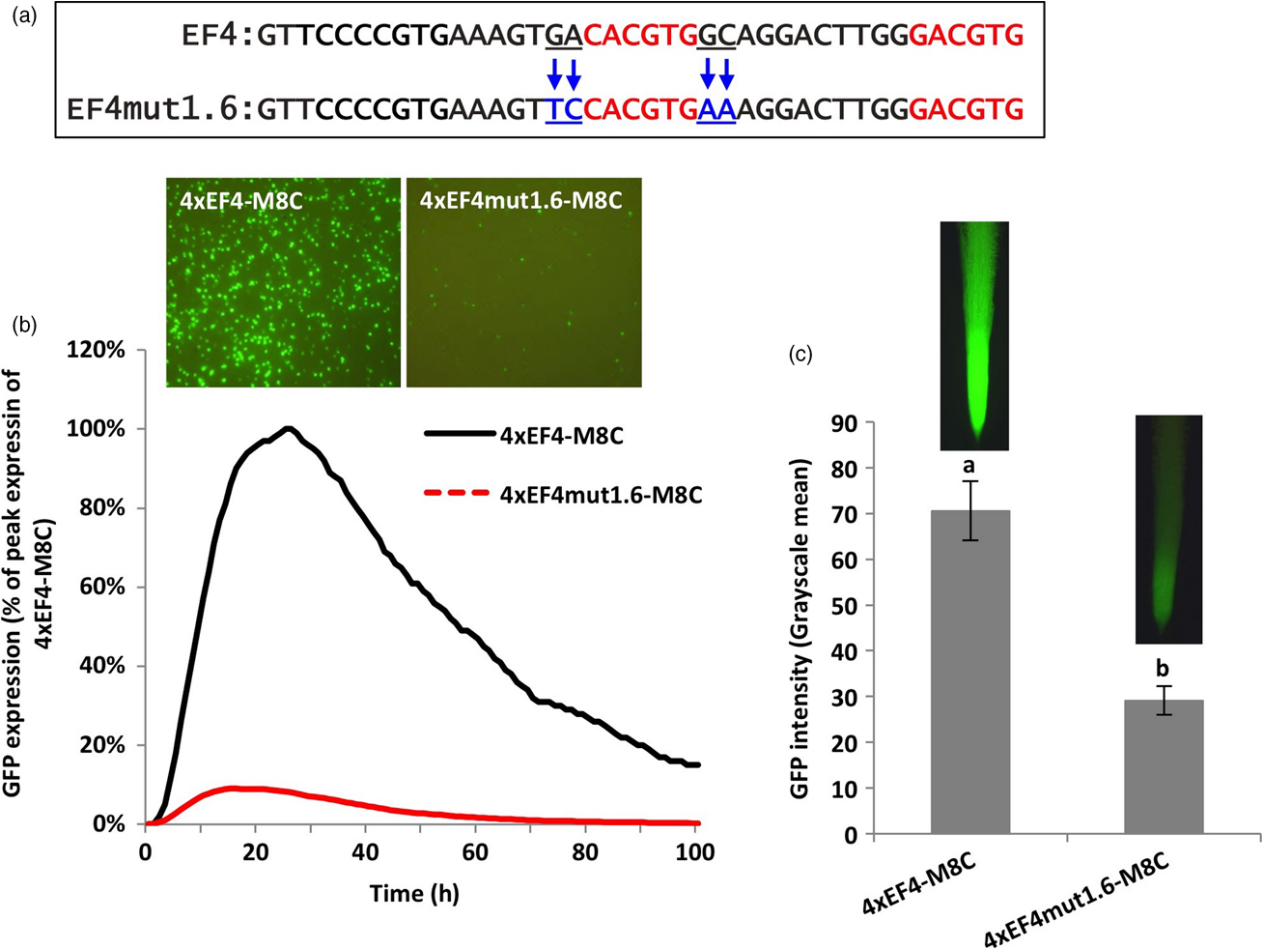
Manipulation of 2‐bp proximal flanks of a G‐box showing decreased gene expression. (a) Mutagenesis of 2‐bp flanking sequences at both sides of the canonical G‐box (CACGTG). (b) Profiles of transient GFP expression in lima bean cotyledon tissues over 100 h. (c) Mean GFP expression intensity (±SE) in soybean hairy roots transformed with different constructs. Columns followed by the same letter are not significantly different by the *t* test (LSD) at *P* < 0.05.

To expand our study beyond the 6‐bp G‐box core sequences, we further evaluated the effects of the flanking sequences on the activity of the 6‐bp G‐box core element. The 2–4 bp flanks of the functional native G‐box and the non‐functional recreated G‐box in the EF4 fragment were therefore interchanged by site‐directed mutagenesis (EF4mut1.5 and EF4mut1.6; Figures 3a,4a). Using transient and stable expression assays, the 4xEF4mut1.5‐M8C construct, where the 2‐bp proximal flanking nucleotides of the non‐functional recreated G‐box were changed to the exact flanking sequences of the functional native G‐box, yielded up to eleven times higher GFP expression in lima bean cotyledonary tissues (Figure 3b) and over twelve times higher GFP expression in stably transformed soybean hairy roots (Figure 3c), compared to the 4xEF4mut1.3‐M8C construct that contained the same recreated G‐box element with its native flanking sequences.

For evaluation of the 4xEF4mut1.6‐M8C in lima bean cotyledons, changes in the 2‐bp proximal flanks of the native classical G‐box element to the native flanks of the recreated G‐box in the EF4 fragment led to decreased levels of GFP expression, which was only 10% of the GFP expression levels driven by the 4xEF4‐M8C (Figure 4b). Similarly, in transformed soybean hairy roots, GFP expression regulated by 4xEF4mut1.6‐M8C was significantly lower compared to the control 4xEF4‐M8C construct (Figure 4c).

**Figure 5:**
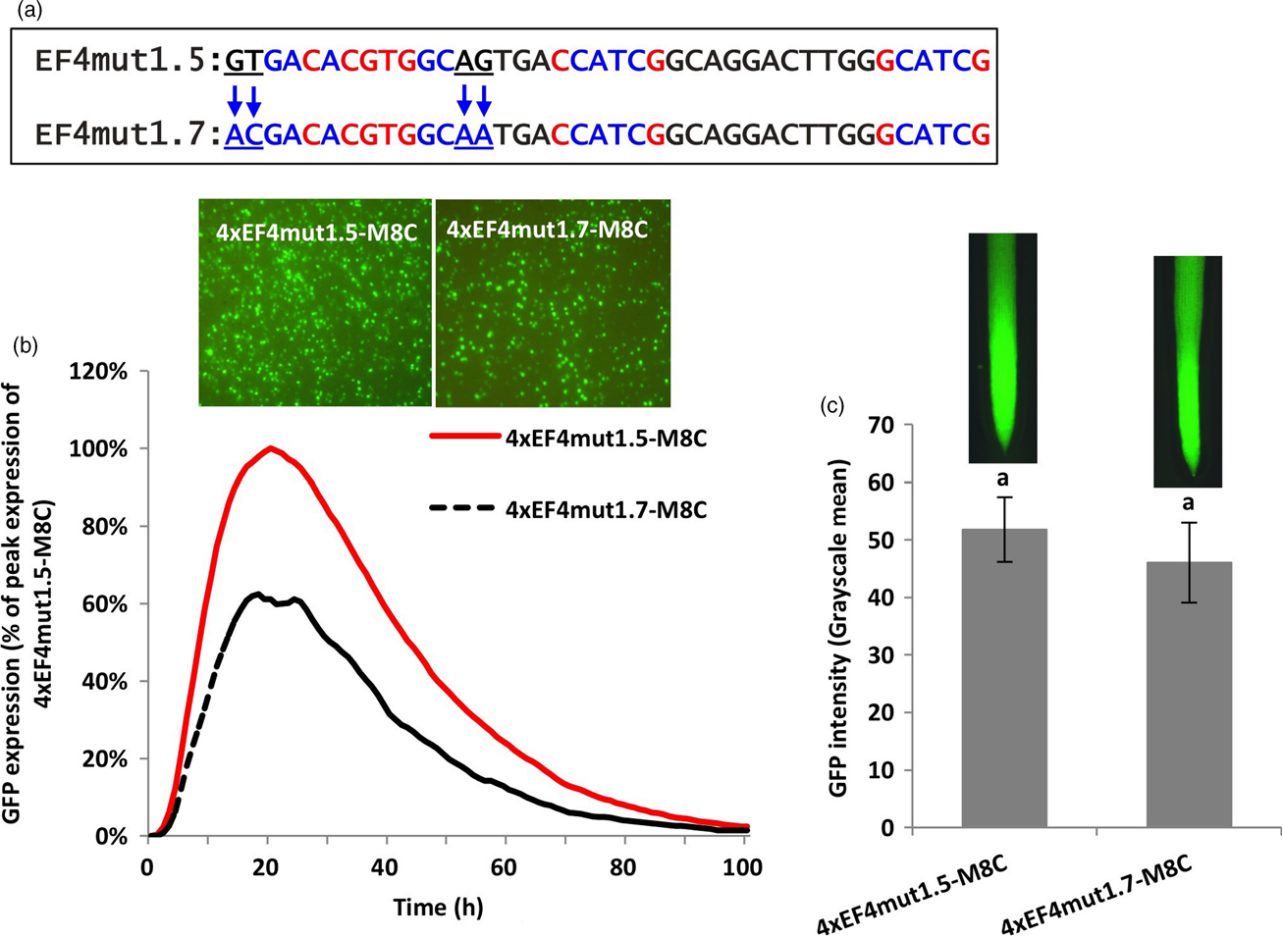
Manipulation of distal and proximal 2‐bp flanks of a G‐box. (a) Mutagenesis of 2‐bp distal flanking sequences of the canonical G‐box (CACGTG). (b) Profiles of transient GFP expression in lima bean cotyledon tissues over 100 h. (c) Mean GFP expression intensity (±SE) in soybean hairy roots transformed with different constructs. Columns followed by the same letter are not significantly different by the *t* test (LSD) at *P* < 0.05.

To investigate the effects of the more distal flanking sequences of the G‐box on gene expression, the third and fourth nucleotides flanking each side of the recreated G‐box were mutated (EF4mut1.7; Figure 5a). The mutant construct 4xEF4mut1.7‐M8C gave about 40% decrease in expression, compared to the 4xEF4mut1.5‐M8C using the transient expression assay (Figure 5b). In stably transformed soybean hairy roots, mutagenesis of the distal flanks in the 4xEF4mut1.7‐M8C only led to slightly lower activity, without a significant difference from the 4xEF4mut1.5‐M8C (Figure 5c).

### Modification of flanking sequences of the G‐box in the GmScream3 (Glycinin) promoter enhanced gene expression

**Figure 6:**
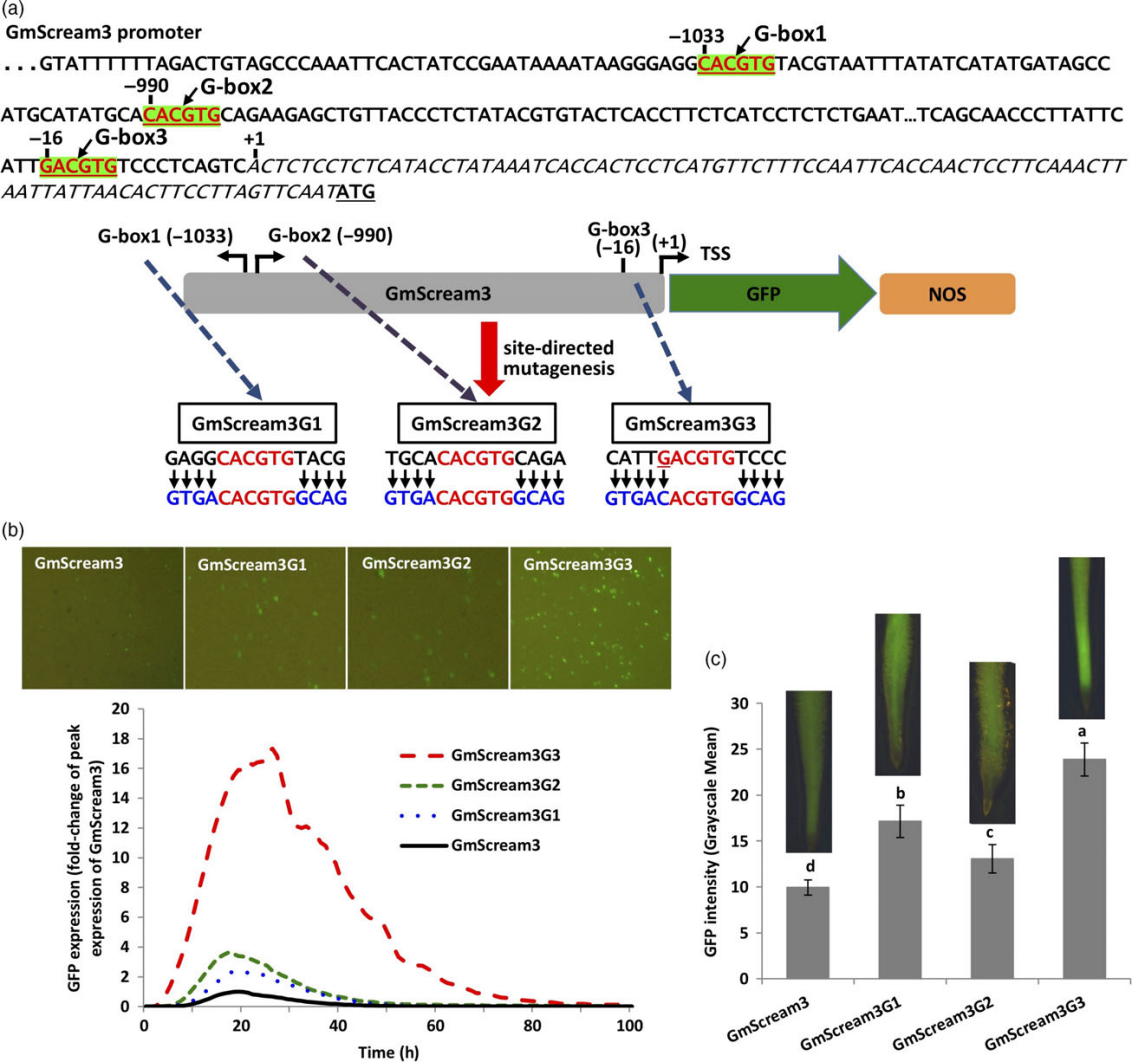
Manipulation of the flanking sequences of three G‐box elements in the native GmScream3 promoter. (a) Position of three G‐box elements (+1 is transcriptional start site) in the GmScream3 promoter and mutagenesis of flanking sequences of each G‐box element. (b) Profiles of transient GFP expression in lima bean cotyledons driven by various GmScream3 mutants over 100 h. (c) Mean GFP expression intensity (±SE) in soybean hairy roots transformed with various GmScream3 mutants. Columns followed by the same letter are not significantly different by the *t* test (LSD) at *P* < 0.05.

Three G‐boxes were identified in the native, relatively weak GmScream3 promoter (1251 bp, Zhang *et al*., 2015; Figure 6), with two classical G‐boxes (G‐box1 and G‐box2; CACGTG) located in the distal region of the promoter and one G‐box variant (G‐box3; GACGTG) located 16‐bp upstream of the TSS (Figure 6a). The 2–4 bp flanking sequences of each G‐box or G‐box variant in the GmScream3 promoter were changed to match the native flanking sequence of the classical G‐box in EF4 (Figure 2a), which was previously demonstrated to confer high gene expression in synthetic promoters using both transient and stable expression assays (Figures 2, 3, 4, 5). Here, modification of the 4‐bp flanking sequences on either side of the two classical G‐boxes in the distal region of the GmScream3 promoter (GmScream3G1 and GmScream3G2, Figure 6a) led to 2.3× and 3.6× higher GFP transient expression in lima bean cotyledons (Figure 6b) and 1.7× and 1.3× higher levels of GFP expression in stably transformed soybean hairy roots respectively (Figure 6c). In comparison, changes in the sequence of the G‐box variant, located in the proximal region of the GmScream3 promoter, into a classic G‐box (CACGTG) along with mutagenesis of its flanking sequences (GmScream3G3, Figure 6a), significantly increased GFP expression up to over 15× higher than the original GmScream3 promoter using transient expression assays (Figure 6b), while the GmScream3G3 also gave rise to 2.4× higher expression than the original GmScream3 promoter in stably transformed soybean hairy roots (Figure 6c).

**Figure 7:**
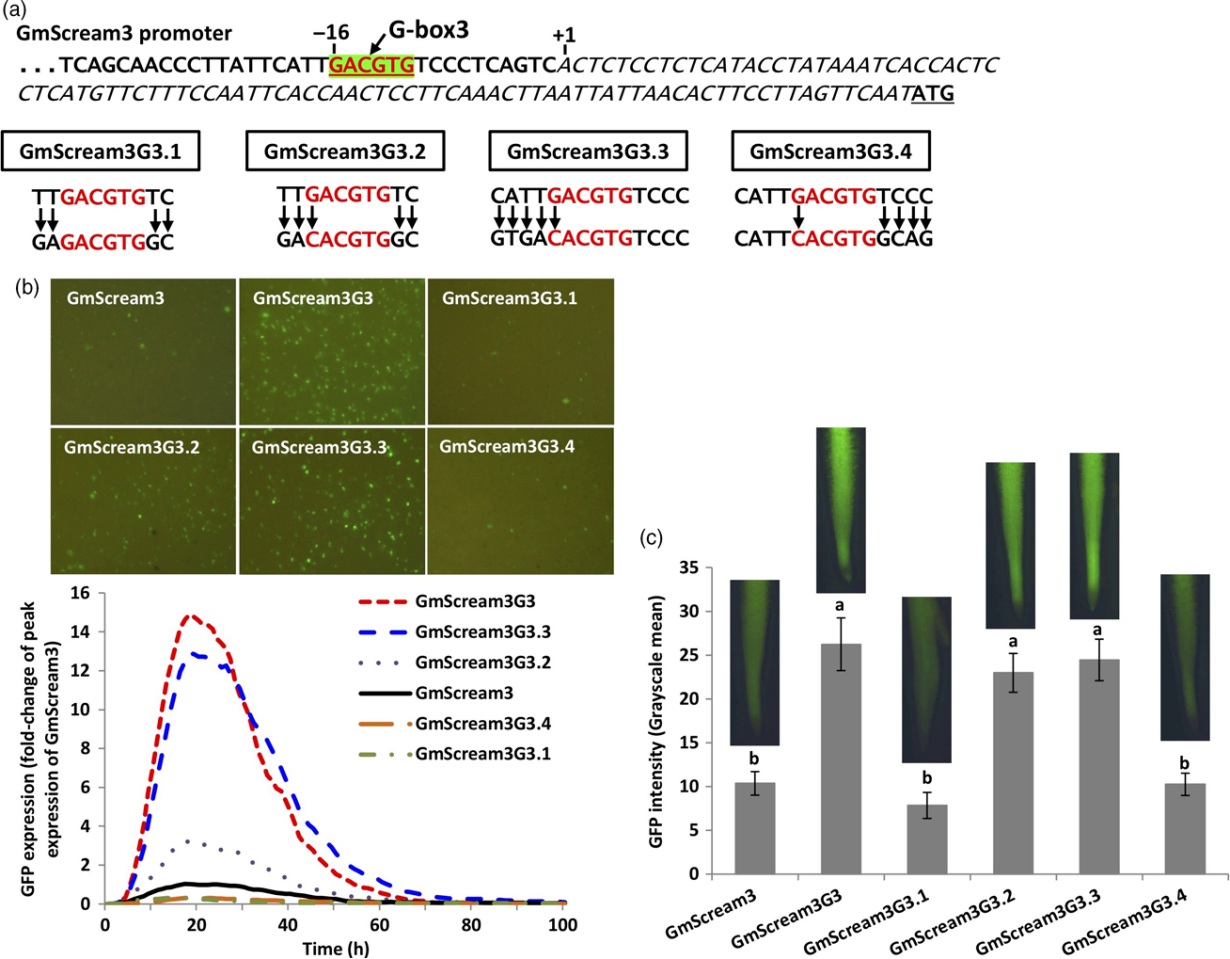
Manipulation of the flanking sequences of the G‐box3 in the GmScream3 promoter. (a) Mutagenesis of flanking sequences of G‐box3 in the GmScream3 promoter. (b) Profiles of transient GFP expression in lima bean cotyledons driven by various G‐box3 mutants over 100 h. (c) Mean GFP expression intensity (±SE) in soybean hairy roots transformed with various G‐box3 mutants. Columns followed by the same letter are not significantly different by the *t* test (LSD) at *P* < 0.05.

To further investigate how the G‐box3 and its flanking sequence affected promoter activity, additional mutants of the G‐box3 in the GmScream3 promoter were generated (Figure 7a). For GmScream3G3.1, only the 2‐bp proximal flanking sequences on both sides of the G‐box3 were mutated to the target nucleotides from the most active G‐box in EF4, while for GmScream3G3.2, in addition to the 2‐bp flanking sequence change, the G‐box3 sequence itself was also converted into the classical G‐box sequence (CACGTG). For the GmScream3G3.3 and GmScream3G3.4 constructs, the G‐box3 variant was mutated into the classical G‐box sequence, and the 4‐bp flanking sequences were mutated into the 4‐bp flanking sequences from the most active EF4 G‐box, either upstream (GmScream3G3.3) or downstream (GmScream3G3.4) of the G‐box (Figure 7a). Among these four additional mutants, the GmScream3G3.3 mutant contributed to the highest levels of GFP expression, comparable to the GmScream3G3, which dramatically augmented promoter activity obtained using both transient expression in lima bean cotyledons and stable expression in soybean hairy roots (Figure 7b–c). The GmScream3G3.2, which contained mutated 2‐bp flanks at each side of a converted classical G‐box, drove threefold higher transient GFP expression in lima bean cotyledons. However, in stably transformed soybean hairy roots, GmScream3G3.2 showed high activity comparable to the GmScream3G3 and GmScream3G3.3 constructs, driving significantly higher GFP expression than the native GmScream3 promoter (Figure 7b–c). In comparison, GFP expression regulated by either GmScream3G3.1 or GmScream3G3.4 was similar to the original native GmScream3 promoter using our two validation tools (Figure 7b–c).

## Discussion

In this study, we identified and characterized a G‐box element with its native flanking sequence from a highly expressing *elongation factor‐1 α* gene (GmScreamM1). In addition, manipulation of the flanking sequences of G‐boxes in a relatively weak soybean glycinin promoter (GmScream3) led to significant increases in the activity of the promoter. This approach of modifying promoter sequences holds great promise for crop improvement by increasing expression of specific genes associated with disease resistance (Hammond‐Kosack and Jones, 1997), photosynthesis (Berry *et al*., 2013), seed storage protein production (Shewry and Halford, 2002) or abiotic stress tolerance (Tester and Bacic, 2005) in soybeans or other crops, by targeted modifications of the native promoter sequences of those target genes using genome editing approaches.

The G‐box was first selected as a candidate element contributing to high activity of several GmScream promoters, as it is a ubiquitous regulatory element in plant promoters (Menkens *et al*., 1995) and can contribute to high levels of gene expression (Ishige *et al*., 1999; McKendree and Ferl, 1992). Use of the plant *cis*‐element database (PlantCARE) for G‐box scanning from our three GmScream promoters identified the classical G‐box sequence (CACGTG) as well as other G‐box variants with differences in the first or last nucleotides of the classical 6‐bp sequence (Figure 1). Evaluation of those putative G‐boxes (CACGTG, GACGTG, TACGTG, CACGTT, AACGTG) (Figure 1a,c) in synthetic promoters using transient expression in lima bean cotyledons and stable expression in soybean hairy roots showed different levels of GFP expression driven by those G‐box‐containing sequences (Figure 1d–e). Although the middle four bases in the six base G‐box core sequence (N*ACGT*N) are generally used for G‐box prediction in promoter sequences by plant *cis*‐regulatory element databases (Higo *et al*., 1999; Lescot *et al*., 2002), our results revealed that G‐boxes with changes in the first and last nucleotides showed significantly lower promoter activity. Plant *cis*‐regulatory element databases are often utilized as element prediction tools, but they cannot be used alone for absolute identification of functional *cis‐*regulatory elements. Validation of putative elements is always needed using conventional promoter deletion analysis followed by transgene introduction and/or targeted mutagenesis approaches as many putative identified elements are not likely functional (Hardison and Taylor, 2012; Hernandez‐Garcia and Finer, 2014).

Using synthetic promoter approaches, the two putative G‐box elements, identified in the EF4 fragment (Figure 2) from the GmScreamM1 promoter, contributed to promoter activity to different degrees. The classical G‐box ‘CACGTG’ significantly enhanced gene expression while the G‐box variant ‘GACGTG’ had little, if any contribution to the high activity of the 4xEF4‐M8C synthetic promoter (Figure 2). Tetrameric repeats of the promoter fragment were evaluated upstream of a promoter core sequence to generate synthetic promoters as this approach has been previously demonstrated to show high sensitivity to small changes in element composition (Hernandez‐Garcia and Finer, 2016; Rushton *et al*., 2002). Changing the sequence of the G‐box variant into a classical G‐box sequence, while mutating the native classical G‐box sequence to make it less functional (4xEF4mut1.4‐M8C, Figure 2) led to a recovery of high GFP expression, indicating that a classical G‐box was able to render high levels of gene expression in either the native EF4 fragment or in the EF4mut1.4 mutant sequence. The classical G‐box and the G‐box variant may have slightly altered functions and may regulate gene expression in different ways, possibly by recruiting different G‐box‐binding transcription factors (Salinas *et al*., 1992; Schindler *et al*., 1992; Slattery *et al*., 2014). On the other hand, the failure to restore high expression by recreating another classical G‐box at a different location in the EF4 fragment (4xEF4mut1.3‐M8C; Figure 2) suggests that the presence of the classical G‐box core element sequence alone in promoters does not guarantee high promoter activity. The contributions of regulatory elements to gene expression strength and specificity may be influenced by element spacing, location within the promoter and proximity to other elements (Liu and Stewart, 2016; Mehrotra *et al*., 2017; Venter, 2007). Further analysis of the flanking sequences of G‐boxes on gene expression (Figures 3, 4, 5) indicated that the proximal flanking sequences of the G‐box had a major effect on G‐box activity, possibly by influencing the specificity and affinity of the GBF binding (Williams *et al*., 1992). This may explain why the native G‐box in the EF4 fragment conferred high levels of gene expression while the recreated G‐box (4xEF4mut1.3‐M8C; Figure 2) showed very weak activity. Protein binding is not necessarily equivalent to gene regulation, and binding affinity is not always a reliable indicator of the effect of TFs on gene expression (Badis *et al*., 2009; Li *et al*., 2008; Slattery *et al*., 2014).

The validation of G‐boxes and their flanking sequences using synthetic promoters indicated that the *cis*‐regulatory element itself could be much larger than the six nucleotide sequences commonly identified using *cis‐*regulatory element databases, with flanking sequences contributing to the intensity and specificity of gene expression regulation. These classically identified 6 bp regulatory elements are ‘core’ element sequences, and the contribution of the flanks and adjacent regulatory elements (Hernandez‐Garcia and Finer, 2016) should be carefully considered when trying to understand promoter functionality. Even though the soybean glycinin promoter (GmScream3) contained two classical G‐boxes and one G‐box variant (Figure 6), this promoter displayed relatively low activity in lima bean cotyledons and hairy roots as we previously reported (Zhang *et al*., 2015). RNAseq revealed that this *glycinin* gene was expressed at very high levels but only in developing seeds (Zhang *et al*., 2015). In addition to the GMScream3 promoter, another glycinin promoter also showed low expression in bombarded lima bean cotyledons and soybean hairy roots while RNAseq revealed high expression in developing seeds (Gunadi *et al*., 2016), as would be expected from seed storage protein genes. These *glycinin* genes were highly expressed but in a tissue specific manner. Manipulation of the flanking sequences of the modified or ‘corrected’ G‐box3 variant in the proximal region of the GmScream3 (glycinin) promoter significantly increased GFP expression using both transient and stable expression assays, while changes of the flanks of the other two classical G‐boxes (G‐box1 and G‐box2) in the far upstream region did not give rise to significant enhancement of gene expression (Figure 6), suggesting that both the flanking nucleotides and the location of the G‐boxes contributed to their functionality in the native promoter. Most functional G‐boxes, which contribute to high levels of gene expression, are often located close to the TSS (Block *et al*., 1990; Faktor *et al*., 1997; McKendree and Ferl, 1992; Sessa *et al*., 1995; Xu and Timko, 2004).

Further mutagenesis of the G‐box3 in the GmScream3 promoter demonstrated that the upstream 2‐4 bp flanks contributed in a large way to the observed gene enhancement (GmScream3G3.3; Figure 7), while the downstream 2–4 bp flanks had little, if any, effect at all (GmScream3G3.4; Figure 7). In addition, in stably transformed soybean hairy roots, the GmScream3G3.2 construct, with only a change in the 2‐bp upstream flanks of the G‐box3 in the Gmscream3 promoter, showed increased activity as high as GmScream3G3 construct (Figure 7). Our results show that gene expression in plants can potentially be greatly enhanced by subtle DNA sequence modification in promoter elements or their flanking sequences. For instance, changes to the two to four flanking nucleotides of G‐box sequences within the promoter sequence, either by genome editing or through spontaneous mutation, may change the expression intensity or specificity of expression of target genes. Although transgene expression can be manipulated by utilizing a selected spatiotemporal or inducible promoter (Bacaj and Shaham, 2007), and the expression of native genes can be controlled by fusing transcriptional activators or repressors to the target promoter guided by catalytically inactive sequence‐specific nucleases (Guan *et al*., 2002; Gupta *et al*., 2012; Lowder *et al*., 2015; Mahfouz *et al*., 2012; Morbitzer *et al*., 2010; Piatek *et al*., 2015), precise modulation of promoter activity by subtle modification of specific DNA motifs holds great promise as no large DNA sequences are introduced (Voytas and Gao, 2014). Genome editing has recently been utilized to generate variants of native promoters with both decreased and increased promoter activity (Rodríguez‐Leal *et al*., 2017). Use of synthetic promoters to evaluate specific mutations to regulatory elements and flanks, as outlined in this study, should be considered as a first step in efforts to modulate native promoter activity using genome editing approaches. Based on our results, it may not be difficult to precisely modify native plant promoters by genome editing of specific regulatory elements and their flanks to obtain altered gene expression.

## Conclusion

We demonstrated that a classical G‐box core sequence (CACGTG) contributed to, but was not alone sufficient for high‐levels of gene expression. The flanking sequences of different G‐boxes significantly affected G‐box activity and promoter activity as shown through the use of both native and synthetic promoters to drive expression of *gfp*. Modification of the 2‐bp proximal flanks of the G‐box in synthetic promoters significantly increased or decreased gene expression over 10‐fold. In addition, modification of the flanking sequences of a G‐box in a soybean glycinin promoter significantly increased the promoter activity using both transient and stable expression assays. Our study indicates the elements are larger than the core, with the contribution of the flanks, which may either provide enhanced strength or specificity.

## Experimental procedures

### Identification of G‐box elements and construction of synthetic promoters

G‐box elements were identified from the three strongest GmScream promoters (GmScreamM1, GmScreamM4 and GmScreamM8; promoters regulate different *elongation factor‐1 α* genes; Zhang *et al*., 2015) using PlantCARE (Lescot *et al*., 2002). The sequence logo of G‐box was created using WebLogo (Crooks *et al*., 2004) by submitting all of the predicted G‐box element sequences. Four promoter fragments, containing those putative G‐box elements together with their flanking sequences, were named EF1, EF2, EF3 and EF4 (EF: Elongation factor‐1 α). Tetramers of the EF1–EF4 fragments were first generated as previously described (Rushton *et al*., 2002). Briefly, upper and lower phosphorylated oligonucleotides of each element‐containing sequence were first annealed together to generate a monomer with *Spe*I and *Xba*I overhangs at the 5′ and 3′ ends respectively. The annealed fragment was then transcriptionally fused to the *green fluorescent protein* (*gfp*) gene in the 35Score‐pFLEV (GenBank accession number: KX814441) or GmScreamM8C‐pFLEV plasmids (GenBank accession number: KX252740) (Zhang *et al*., 2016). The 35Score‐pFLEV or GmScreamM8C‐pFLEV constructs contain a *gfp* gene regulated by the 46‐bp 35S core promoter or the 100‐bp GmScreamM8 core promoter with a native GmScreamM8 leader intron respectively. Plasmids containing the EF1‐EF4 monomer were separately digested with *Spe*I/*Eco*RI and *Xba*I/*Eco*RI. The larger fragment from *Xba*I/*Eco*RI digestion and the smaller fragment from *Spe*I/*Eco*RI digestion were then gel purified and subsequently lighted to double the copy number of the element fragment, retaining only a pair of *Spe*I/*Xba*I restriction sites outside of the dimeric element fragment. Repeating the above digestion‐ligation step with the dimerized element constructs generated the synthetic tetrameric element constructs.

To generate a series of element mutants containing either a modified G‐box in the EF4 fragment (EF4mut1, EF4mut1.1, EF4mut1.2, EF4mut1.3, EF4mut1.4, Figure 2a) or changes to the 2–4 bp flanking sequences outside of the G‐box (EF4mut1.5, EF4mut1.6, EF4mut1.7, Figures 3a,4a,5a), the same approach mentioned above was used to generate synthetic promoters containing tetrameric element mutants with the modification to the targeted DNA sequences in the synthesized oligonucleotide sequences.

All the element‐containing pFLEV plasmids were transformed into *Escherichia coli* DH5α by heat shock and their identity was confirmed by DNA sequencing (Eurofins, Louisville, KY). To subclone the whole expression cassettes into the binary expression vector pCAMBIA1300 (CAMBIA, Canberra, Australia) for generation of soybean hairy roots, the synthetic promoters, the *gfp* coding region and the *NOS* terminator were excised as an intact unit from pFLEV following digestion with the appropriate restriction enzymes and subcloned into the multiple cloning site (MCS) of pCAMBIA1300.

### Inverse PCR

To construct a series of mutants in the flanking and/or core sequences of the G‐box elements in the GmScream3 (Glycinin) promoter (Zhang *et al*., 2015) to generate GmScream3G1, GmScream3G2, GmScream3G3, GmScream3G3.1, GmScream3G3.2, GmScream3G3.3 and GmScream3G3.4 (Figures 6, 7), inverse PCR was performed as previously described (Xu and Gong, 1999) with some modifications. Two inverted tail‐to‐tail primers were designed with site‐targeted mutations to amplify the entire plasmid sequence (Table S1). PCR was performed using a FailSafe™ PCR Kit (Epicenter Biotechnologies, Madison, WI) according to the manufacturer's instructions using the original GmScream3‐pFLEV plasmid (GenBank accession number: KX252719) as template. After a 1 μl aliquot of PCR product was electrophoresed in a 1% agarose gel to check the size of the amplicon, PCR products were purified using DNA clean & concentrator™‐5 kit (Zymo Research, Irvine, CA), followed by 3′ blunting and 5′ phosphorylation using End‐it™ DNA End‐Repair Kit (Epicenter Biotechnologies). The purified PCR products were then treated with *Dpn*I at 37 °C for 1 h to remove the methylated parental non‐mutated plasmid DNA and subsequently self‐ligated using a Quick ligation kit (NEB, Ipswich, MA). The ligated plasmids were transformed into *E. coli* DH5α by heat shock. Plasmid DNA was isolated from PCR‐positive single colonies and sequenced to select mutants with the correctly modified flanking and/or core sequences of the G‐boxes in the promoter constructs.

### Gene expression quantification and data analysis

All promoter/element constructs were evaluated using transient expression in lima bean cotyledons as previously described (Zhang *et al*., 2015). In brief, lima bean (*Phaseolus lunatus* cv ‘Henderson Bush’) seeds were sterilized with 4% (v/v) bleach and germinated in GA7 culture boxes with moistened paper towels. Cotyledons were excised from 4‐day‐old germinating seedlings, placed on a medium containing Murashige and Skoog salts, (Murashige and Skoog, 1962), B5 vitamins (Gamborg *et al*., 1968), 3% sucrose and 0.2% Gelrite (Sigma‐Aldrich, St. Louis, MO) without plant regulators (OMS medium) at pH 5.7. Cotyledons were then placed on a stainless steel mesh supporting screen with the adaxial surface up, and bombarded with tungsten particles coated with different DNA constructs using the Particle Inflow Gun (Finer *et al*., 1992). Bombarded cotyledons were placed in Petri dishes, which were covered with thick sterile polycarbonate lids to minimize condensation (Finer and Finer, 2007). Dishes were mounted on a custom‐designed, computer‐controlled, 2‐dimensional robotics platform positioned under a MZFLIII dissecting fluorescence microscope (Leica, Heerbrugg, Switzerland), and images of each cotyledon were collected every hour for 100 h (Chiera *et al*., 2007; Hernandez‐Garcia *et al*., 2010b). Captured images displaying different levels of GFP expression driven by different promoter/element constructs were analysed as previously described (Hernandez‐Garcia *et al*., 2010b). Each image series (100 images) for each construct was first manually aligned using Adobe ImageReady to make sure that the same GFP‐expressing area in the 100 images was analyzed. A 300 × 400 pixel area of each aligned image series was then selected and used for GFP intensity quantification using ImageJ (Rasband, 1997). Batch images were separated into red, green, and blue channels, and background gray values, obtained from a non‐GFP expressing region of the cotyledon, were subtracted from each image at each time point. The background‐corrected GFP intensity was then calculated by multiplying the mean grayscale value per pixel in the red and green channels by the total number of GFP‐expressing pixels in each channel. GFP expression was then presented as the percentage or fold‐change of the peak expression of a control promoter/element construct. For each construct, three cotyledons were bombarded for each experiment, with two or three independent biological replications, generating six to nine repetitions per construct for transient expression analysis.

Stably transformed soybean hairy roots containing various promoter/element constructs were also generated and used for GFP expression analysis as described by Hernandez‐Garcia *et al*. (2010a). In brief, soybean (*Glycine max* cv ‘Williams82’) seeds were sterilized and germinated as described above for the lima bean seeds. After 6 days, cotyledons were excised and inoculated with *Agrobacterium rhizogenes* K599 containing different promoter/element constructs. After 3 days, cotyledons were transferred to OMS medium containing 400 mg/L Timentin to control growth of the *Agrobacterium*. After an additional 2–3 weeks, hairy roots were excised and transferred to fresh OMS medium containing 400 mg/L Timentin and 25 mg/L hygromycin. After 4 days, images of GFP‐expressing hairy roots (~2 cm) were collected using the MZFLIII dissecting microscope equipped with a GFP2 filter set (Ex. 480 ± 40 nm; Em. 510 nm), and a Spot‐RT CCD digital camera (Diagnostic Instruments Inc., Sterling Heights, MI). GFP intensity was measured using ImageJ as previously described (Hernandez‐Garcia *et al*., 2010a). In brief, images of individual roots were separated into red, green and blue channels, and GFP intensity was measured by calculating the background‐corrected grayscale mean value using only the green channel. The final GFP expression values for each promoter/element construct were calculated by subtracting the grayscale mean value of hairy roots induced by *A. rhizogenes* without the binary vector from an average value for the GFP‐expressing hairy roots. For each construct, at least 20 transgenic events were generated and analyzed, with at least two independent replications. Comparisons between different constructs were analyzed using one‐way ANOVA. The significant difference between the means was analyzed using the Student's *t* test (LSD) at *P* < 0.05.

## Conflict of interest

The authors declare that they have no conflict of interest.

## Author contributions

NZ, JJF, and LKM conceived and designed the experiments. NZ designed and constructed the vectors, and performed the DNA introductions. NZ and JJF performed the analysis of the collected images and data. NZ, JJF and LKM interpreted the data and wrote the manuscript.

## Supporting information


**Table S1** List of primer sequences used for PCR amplification. F‐forward primer, R‐reverse primer
