# Supplementary material for: Changes to the core and flanking sequences of G‐box elements lead to increases and decreases in gene expression in both native and synthetic soybean promoters
Source: Plant Biotechnol J. 2018 Sep 24;17(4):724–35. doi: 10.1111/pbi.13010 (PMC6419578; doi:10.1111/pbi.13010)
Supplement: Supplementary file 1 — Table S1 List of primer sequences used for PCR amplification. F‐forward primer, R‐reverse primer [file PBI-17-724-s001.docx]

**Supplementary Table 1. List of primer sequences used for PCR amplification**

F-forward primer, R-reverse primer.

| Promoter | Primer sequence (5’ to 3’) |
| --- | --- |
| EF1 | TCTAATCCGAACTACGAGACGTGAGAAGCACGCGCTTTAG |
| EF2 | ATCCTTTTACGTGTGCTGTGAGACATTATCATCAATTGTG |
| EF3 | CCAGCATTTGCCACGTTTGAACGTGAGCCGAAACGATGTC |
| EF4 | GTTCCCCGTGAAAGTGACACGTGGCAGGACTTGGGACGTG |
| EF4mut1 | GTTCCCCGTGAAAGTGACCATCGGCAGGACTTGGGCATCG |
| EF4mut1.1 | GTTCCCCGTGAAAGTGACCATCGGCAGGACTTGGGACGTG |
| EF4mut1.2 | GTTCCCCGTGAAAGTGACACGTGGCAGGACTTGGGCATCG |
| EF4mut1.3 | GTTCCACGTGAAAGTGACCATCGGCAGGACTTGGGCATCG |
| EF4mut1.4 | GTTCCCCGTGAAAGTGACCATCGGCAGGACTTGGCACGTG |
| EF4mut1.5 | GTGACACGTGGCAGTGACCATCGGCAGGACTTGGGCATCG |
| EF4mut1.6 | GTTCCCCGTGAAAGTTCCACGTGAAAGGACTTGGGACGTG |
| EF4mut1.7 | ACGACACGTGGCAATGACCATCGGCAGGACTTGGGCATCG |
| GmScream3G1 | F: GCAGTAATTTATATCATATGATAGCCATGC |
|  | R: CACGTGTCACCCTTATTTTATTCGGA |
| GmScream3G2 | F: GCATATGTGACACGTGGCAGAAGAGCTGTTAC |
|  | R: ATGGCTATCATATGATATAAATTACGTACACG |
| GmScream3G3 | F: GCAGTCAGTCACTCTCCTCTCATACCTA |
|  | R: CACGTGTCACAATAAGGGTTGCTGAGTTTG |
| GmScream3G3.1 | F:GCCCTCAGTCACTCTCCTCTCATACCTA |
|  | R:CACGTCTCTGAATAAGGGTTGCTGAGTTTG |
| GmScream3G3.2 | F:GCCCTCAGTCACTCTCCTCTCATACCTA |
|  | R:CACGTGTCTGAATAAGGGTTGCTGAGTTTG |
| GmScream3G3.3 | F:TCCCTCAGTCACTCTCCTCTCATACCTA |
|  | R:CACGTGTCACAATAAGGGTTGCTGAGTTTG |
| GmScream3G3.4 | F:GCAGTCAGTCACTCTCCTCTCATACCTA |
|  | R:CACGTGAATGAATAAGGGTTGCTGAGTTTG |
